# A unified spatiotemporal–geometry framework for target classification and localisation in dual-static passive radar

**DOI:** 10.1371/journal.pone.0350515

**Published:** 2026-06-02

**Authors:** Hongmin Wang, Zhiyong Lei, Xing Liu

**Affiliations:** 1 School of Mechatronic Engineering and Engineering Training Center, Xi’an Technological University, Xi'an, Shaanxim, China; 2 Engineering Training Center, Xi'an Technological University, Xi'an, Shaanxi, China; 3 School of Electronic and Information Engineering, Xi’an Technological University, Xi'An, Shaanxi, China; Northwest Normal University, CHINA

## Abstract

Passive radar exploits ambient broadcast signals and requires no dedicated transmitter, making it attractive for covert surveillance and target monitoring. A fundamental difficulty arises at low signal-to-noise ratio (SNR) or when targets move slowly: the class decision (static vs. dynamic) and the geometry-based position estimate are solved in two independent steps by most existing methods, which can lead to inconsistent outputs. We propose a joint spatiotemporal–geometry framework for a dual-static passive radar operating on DVB-T broadcast signals at 650 MHz. The framework combines a spatiotemporal encoder with dilated convolutions and cross-attention, and a Cramér–Rao-weighted Levenberg–Marquardt bistatic solver. The two components are coupled through an iterative optimisation loop: the encoder class probability steers a physics-consistent velocity penalty inside the solver, while the updated solver state feeds back into the next class decision. Unlike prior joint methods that either operate on sequential tracks or incorporate physics only at training time, the proposed framework enforces the exact bistatic delay and Doppler equations as hard constraints at every test-time iteration while the encoder class probability actively steers the geometry penalty within the same optimisation loop. Across 500 Monte Carlo trials per SNR point and five independent evaluation seeds, the proposed method achieves a mean classification accuracy of 93.7 ± 0.8% with a weighted F1-score of 0.937 ± 0.007. The mean localisation error at −6 dB SNR is 1.15 ± 0.09 km, a 28.1% reduction compared with a geometry-only baseline. The joint optimisation converges in a mean of 4.1 ± 0.8 outer iterations. A sensitivity analysis confirms that all results are stable across a factor-of-two variation in any single hyperparameter. Within the simulated dual-static passive radar environment considered in this study, the proposed iterative approach consistently outperforms seven evaluated baseline methods in both classification accuracy and localisation error.

## Introduction

Passive radar uses broadcast signals such as DVB-T or FM radio instead of sending its own signal [1–3]. Because of this, passive radar does not need a dedicated transmitter and does not reveal its own presence. These properties make passive radar attractive for surveillance and target monitoring. In the last decade, many researchers have worked on passive radar for target detection, classification, and localisation.

A fundamental difficulty is that the received echo is often weak, especially when the target is small or moves slowly. At low SNR, the delay and Doppler estimates from the cross-correlator become noisy and the localisation error becomes large [4,5]. A further complication is that slowly moving targets produce only a small bistatic Doppler shift of a few hertz, which is hard to separate from clutter [6]. Classical geometry methods do not address this well because they depend on clean delay and Doppler measurements.

Deep learning methods have shown strong results for radar target classification. Several studies use convolutional neural networks (CNN) and recurrent networks to learn temporal patterns from micro-Doppler features [7–9]. However, most of these methods do not incorporate a geometry constraint, so the class output can be inconsistent with the actual bistatic measurement. To make this concrete: if the encoder outputs “static” with probability 0.61 for a slow-moving target at −6 dB SNR, the non-iterative variant applies the static velocity penalty and collapses the velocity estimate to near zero, producing a position error of 2.1 km. The proposed iterative method revises the class to “dynamic” by iteration 2 and recovers a 1.2 km position error—a 43% improvement on this challenging case (see also Section).

Algorithm unrolling connects classical signal processing with deep learning [10]. Attention and transformer mechanisms have also been applied to radar classification tasks [11,12], showing that temporal attention can improve performance. But these methods still treat classification and localisation as separate problems.

The key gap in the literature is that no existing method for dual-static passive radar actively enforces consistency between the class decision and the geometry estimate at every test-time iteration. Some recent work begins to address this by combining learning and geometry [13–15], but these works either use a one-step coupling or do not apply the exact bistatic geometry equations as hard constraints. Yu et al. [16] adopt iterative tracking-and-classification but operate on monostatic sequential tracks and do not use bistatic delay–Doppler as hard constraints. In contrast, our method uses an iterative loop in which the encoder and the solver update each other at every step, with the bistatic equations enforced as hard constraints throughout.

The main contributions of this paper are:

- We propose a joint spatiotemporal–geometry framework for dual-static passive radar in which the exact bistatic delay and Doppler equations are enforced as hard constraints at every test-time iteration, and the encoder class probability actively steers the geometry penalty within the same optimisation loop. To the best of our knowledge, this specific instantiation for the bistatic passive radar measurement model has not been previously reported.
- We design a lightweight spatiotemporal encoder (47,362 parameters) using dilated convolutions and cross-attention that provides a calibrated class probability to the geometry solver across *N* = 20 coherent processing intervals (CPIs).
- We derive a class-dependent velocity penalty from a Bayesian MAP regularisation of the velocity estimate under a Laplacian prior, and embed it into a Cramér–Rao-weighted Levenberg–Marquardt solver for bistatic passive radar.
- We provide a comprehensive evaluation including sensitivity analysis, ablation at −6 dB SNR, generalisation tests across SNR and baseline, and comparison with seven baselines including CRLB-weighted geometry, an unrolled solver, a transformer-based end-to-end model, and an indicative re-implementation of the method of Yu et al. [16].

The remainder of the paper is organised as follows. The next section reviews related work. We then describe the system model and proposed method, followed by the simulation and training setup. Results and discussion are presented together, with sub-sections covering classification, localisation, ablation, convergence, Doppler consistency, sensitivity analysis, generalisation analysis, and limitations. The final section concludes.

## Related work

### Classical bistatic geometry methods

Griffiths and Baker [1] describe passive coherent location systems and show that geometry methods work well at high SNR. Malanowski and Kulpa [4] compare localisation methods for multistatic passive radar. Ho and Xu [5] give an algebraic solution for moving-source location using TDOA and FDOA measurements, showing that localisation error is highly sensitive to measurement noise. Howland et al. [2] study FM-based bistatic radar and show that broadcast signals can cause delay estimation errors. These classical works confirm that geometry methods are effective at high SNR but provide no mechanism to leverage learning when conditions are poor.

### Deep learning for radar classification

Gurbuz and Amin [7] show that deep networks give strong results for human motion recognition from radar Doppler features. Kim and Moon [8] classify targets from micro-Doppler images using a CNN. Clemente et al. [9] review micro-Doppler signatures for classification. Geng et al. [12] survey deep learning for radar and identify the separation of classification and physics-based estimation as a common limitation. Ritchie et al. [6] classify micro-drones in a multistatic passive radar system.

### Attention and transformer methods

Vaswani et al. [17] introduced the attention mechanism that underlies transformer architectures. Wu et al. [11] apply attention networks to radar human activity recognition and show improved accuracy at low SNR. Yu et al. [16] propose a joint tracking and classification approach for manoeuvring sources; they adopt an iterative bidirectional coupling between a classifier and a physics-based tracker, which is conceptually related to our framework. However, their method is designed for monostatic radar and operates on sequential tracks via a Kalman filter; it does not use bistatic range or Doppler as hard constraints, nor does it enforce the bistatic delay–Doppler equations at each test iteration as we do.

### Algorithm unrolling and physics-informed networks

Monga et al. [10] survey algorithm unrolling for signal and image processing. Papageorgiou et al. [18] apply a deep network for direction-of-arrival estimation at low SNR and show that physics knowledge can be built into the network structure. Temiz et al. [19] study improved localisation in a hybrid multistatic radar network. Colone et al. [20] review passive radar challenges and data-driven approaches.

### End-to-end deep learning for localisation

Zhang et al. [13] use an encoder–decoder network to estimate target position from range-Doppler maps. Xu et al. [14] apply a physics-informed neural network (PINN) for TDOA-based localisation, adding a delay residual penalty to the loss. Wang et al. [15] propose an end-to-end trainable network for joint position and class estimation in monostatic radar. These end-to-end methods typically require large labelled training sets and do not apply the bistatic geometry equations as hard constraints at test time.

### Summary and motivation

The separation of geometry-based localisation and learning-based classification is a recurring limitation in the literature. While several methods adopt some form of coupling between learning and geometry [14–16], they differ from our approach in key ways: (i) methods such as [16] use iterative coupling but operate on sequential monostatic tracks without bistatic constraints; (ii) methods such as [14,15] embed physics in the training loss but do not enforce it at test time. In contrast, we use an iterative feedback loop so that the class estimate influences the solver penalty and the solver output influences the next class decision, while (ii) we use the exact bistatic delay and Doppler equations from [1,4,5] as hard constraints at every test iteration. The encoder augments the geometry with a class prior rather than replacing it.

## Materials and methods

### Dual-static passive geometry

We consider a dual-static passive radar with one transmitter of opportunity at fixed position **T** and two static receivers at positions **R**_1_ and **R**_2_. The target is at position **x**(*t*) with velocity **v**(*t*). Both receivers collect the target echo. The transmitter and receiver positions are assumed known; the system is synchronised in time and frequency via the direct path signal.

For receiver *i*, the bistatic range is


$$\begin{array}{c} \rho_{i}(𝐱)=\|𝐱-𝐓\|+\|𝐱-{𝐑}_{i}\|-\|𝐓-{𝐑}_{i}\|, \end{array}$$ (1)

and the bistatic delay is


$$\begin{array}{c} \tau_{i}(𝐱)=\rho_{i}(𝐱)/c. \end{array}$$ (2)

The bistatic Doppler shift for a target with velocity **v** is


$$\begin{array}{c} f_{i}(𝐱,𝐯)=\frac{1}{\lambda }\left[𝐯\cdot \frac{𝐓-𝐱}{\left\|𝐓-𝐱\right\|}+𝐯\cdot \frac{{𝐑}_{i}-𝐱}{\left\|{𝐑}_{i}-𝐱\right\|}\right], \end{array}$$ (3)

where the unit vectors point from the target toward the transmitter and receiver, respectively. For a static target (𝐯=0), Eq (3) gives zero Doppler [1]. The noisy measurement vector after cross-correlation is


$$\begin{array}{c} 𝐦=[{\tilde{\tau }}_{1},\;{\tilde{\tau }}_{2},\;{\tilde{f}}_{1},\;{\tilde{f}}_{2}{]}^{⊤}. \end{array}$$ (4)

In 2D, the four unknowns (*x*, *y*, *v*_*x*_, *v*_*y*_) are matched by four measurements. Two bistatic ellipses can intersect at two points (ghost-target ambiguity); we resolve this by choosing the intersection whose predicted Doppler from Eq (3) has the smallest residual with the measured Doppler [5]. When the measured Doppler is near zero (i.e., $|{\tilde{f}}_{i}|<MDV$) at very low SNR, the Doppler discriminant becomes unreliable; in that case the system falls back to the intersection with the smaller geometry cost *J*_geo_, as noted in the Limitations section. Across 2,000 test samples at −6 dB SNR, the Doppler discriminant is reliable in 94.1% of cases; the geometry-cost fallback is triggered in the remaining 5.9%. In the fallback cases, the ghost-selection error rate is 8.3%, compared with 3.1% when the Doppler discriminant is used. This confirms that low-SNR ghost resolution is a genuine source of localisation error and motivates the future integration of a multi-hypothesis tracker to resolve ambiguity over consecutive snapshots.discussed further in the Limitations

### Signal model and feature extraction

Each receiver has a reference channel and a surveillance channel. The surveillance signal is


$$\begin{array}{c} r_{s}(t)=\alpha (t)\;s(t-\tau (t))\;\exp\;(j2\pi f(t)t)+n(t). \end{array}$$ (5)

The cross-ambiguity function (CAF) per CPI is


$$\begin{array}{c} A_{i}(\tau ,f;n)=\int r_{s,i}(t)\;r_{ref,i}^{*}(t-\tau )\;\exp(-j2\pi ft)\;w_{n}(t)\;dt. \end{array}$$ (6)

From the CAF peak for each receiver and CPI, we extract five features: estimated delay ${\tilde{\tau }}_{i}(n)$, estimated Doppler ${\tilde{f}}_{i}(n)$, peak amplitude *a*_*i*_(*n*), peak width $\sigma_{i}(n)$, and local energy *e*_*i*_(*n*). Over *N* = 20 intervals and two receivers, the feature sequence is $ℋ=\{{𝐲}_{1}(n),{𝐲}_{2}(n){\}}_{n=1}^{N}$. All features are normalised by *z*-score over the training set.

### Spatiotemporal encoder

The spatiotemporal encoder converts ℋ into a global descriptor $\bar{𝐳}$. The encoder is intentionally lightweight (47,362 parameters total) because its role is to provide a calibrated class prior to the geometry solver, not to replace it. It uses three dilated convolution layers [21] to capture patterns at multiple time scales, followed by a four-head cross-attention module [17] to learn which CPI intervals are most informative. Table 1 shows the full architecture.

**Table 1:** Spatiotemporal encoder architecture. All parameter counts are exact and verified against the implementation.

Layer	Type and configuration	Output	Params
Input	Feature sequence (*N* = 20, 10 features)	*N* × 10	—
Conv-1	Dilated Conv1D, rate = 1, 64 filters, *k* = 3, ReLU	*N* × 64	1,984
Conv-2	Dilated Conv1D, rate = 2, 64 filters, *k* = 3, ReLU	*N* × 64	12,352
Conv-3	Dilated Conv1D, rate = 4, 64 filters, *k* = 3, ReLU	*N* × 64	12,352
Cross-Attn	4-head self-attention, *d*_model_=64	*N* × 64	16,384
GlobalPool	Average pooling over *N* time steps	64	—
FC	Fully connected, 64→64, ReLU	64	4,160
Classifier	Fully connected, 64→2, Softmax	2	130
Total			47,362

We also tested larger variants with 128 and 256 filters (179,970 and 718,722 parameters respectively). Accuracy improved marginally to 94.1% and 94.3%, while CPU inference time increased from 0.8 ms to 3.1 ms and 11.4 ms per sample. We retain 64 filters as the best accuracy-to-complexity trade-off for near-real-time embedded deployment alongside the LM solver.

The per-interval vectors and global descriptor are


$$\begin{array}{c} \{𝐳(n){\}}_{n=1}^{N}=f_{enc}(ℋ;\theta ),\;\bar{𝐳}=\frac{1}{N}\sum_{n=1}^{N}𝐳(n). \end{array}$$ (7)

The encoder and classifier are trained jointly by minimising cross-entropy over *M* training samples:


$$\begin{array}{c} ℒ=-\frac{1}{M}\sum_{m=1}^{M}\log p(c^{m}|{\bar{𝐳}}^{m}). \end{array}$$ (8)

### Geometry-based localisation

To estimate position **x** and velocity **v**, we minimise a weighted geometry cost


$$\begin{array}{c} J_{geo}(𝐱,𝐯)=\sum_{i}w_{\tau ,i}({\tilde{\tau }}_{i}-\tau_{i}(𝐱){)}^{2}+\sum_{i}w_{f,i}({\tilde{f}}_{i}-f_{i}(𝐱,𝐯){)}^{2}, \end{array}$$ (9)

with Cramér–Rao-derived weights


$$\begin{array}{c} w_{\tau ,i}=\frac{8\pi^{2}B^{2}\rho_{i}T}{3},\;w_{f,i}=\frac{2\pi^{2}T^{2}\rho_{i}}{3}, \end{array}$$ (10)

where $\rho_{i}$ is the linear SNR at receiver *i* and *T* is the CPI duration. These weights are derived from the diagonal entries of the Fisher Information Matrix (FIM) of the bistatic measurement model under Gaussian noise (see Supplementary Note S1 for the full derivation).

### Supplementary Note S1: Derivation of CRLB-based weighting (summary)

The Fisher Information Matrix (FIM) for the bistatic measurement vector $𝐦=[{\tilde{\tau }}_{1},{\tilde{\tau }}_{2},{\tilde{f}}_{1},{\tilde{f}}_{2}{]}^{⊤}$ under independent Gaussian noise with variance $\sigma_{\tau ,i}^{2}=3/(8\pi^{2}B^{2}\rho_{i}T)$ for delays and $\sigma_{f,i}^{2}=3/(2\pi^{2}T^{2}\rho_{i})$ for Doppler [4,5] is diagonal with entries $[𝐅{]}_{ii}=1/\sigma_{i}^{2}$. The optimal weighted least-squares cost uses weights $w_{i}=1/\sigma_{i}^{2}$, which directly yields Eq. (10). The full position-space CRLB is $CRLB(𝐱)=[{𝐉}^{⊤}𝐅𝐉{]}^{-1}$, where **J** is the Jacobian of the measurement vector with respect to (**x**, **v**). The scalar CRLB plotted in Table 6 is the square root of the trace of the position block of this matrix, evaluated at the representative mid-field geometry.

Compared with uniform weighting, CRLB-weighting allocates more influence to high-SNR measurements and reduces localisation error by approximately 11.8% at 0 dB SNR (see Table 7).

A class-dependent velocity penalty is


$$\begin{array}{c} \kappa (c,𝐯)=\left\{ \begin{array}{cc} \lambda_{s}\|𝐯{\|}^{2} & c=0\text{ (static)},\\ \lambda_{d}\max(0,\;v_{\min}-\|𝐯\|{)}^{2} & c=1\text{ (dynamic)}, \end{array}\right. \end{array}$$ (11)

This penalty can be motivated from a Bayesian MAP perspective: assuming a zero-mean Laplacian prior on **v** for static targets and a lower-truncated Laplacian prior for dynamic targets, the MAP estimate of **v** given the class *c* leads exactly to the quadratic penalty in Eq. (11). The coefficients $\lambda_{s}$ and $\lambda_{d}$ correspond to the inverse prior variance and are set empirically ($\lambda_{s}=1.0$, $\lambda_{d}=2.0$); a sensitivity analysis is provided in Section. The total localisation cost is


$$\begin{array}{c} J_{loc}(𝐱,𝐯;c)=J_{geo}(𝐱,𝐯)+\kappa (c,𝐯). \end{array}$$ (12)

### Joint iterative optimisation

The full joint cost connects the encoder and the solver:


$$\begin{array}{c} J_{tot}(𝐱,𝐯,c)=J_{geo}(𝐱,𝐯)+\kappa (c,𝐯)-\gamma \log p(c|\bar{𝐳}). \end{array}$$ (13)

The term $-\gamma \log p(c|\bar{𝐳})$ biases the solver toward the class the encoder assigns a higher probability. The weight γ controls the encoder influence. Iteration stops when $|\Delta J_{tot}|<10^{-3}$ or when the iteration count reaches $K_{\max}=10$.

The procedure is:

1. Compute $\bar{𝐳}=f_{enc}(ℋ;\theta )$ and $p(c|\bar{𝐳})$ from the classifier.
2. Set $c^{\left(0\right)}=\text{arg}\max_{c}\;p(c|\bar{𝐳})$.
3. Initialise (**x**^(0)^, **v**^(0)^) from the bistatic ellipse intersection; resolve ghost ambiguity via Doppler [5].
4. For $k=0,1,\ldots ,K_{\max}$:
  a) $\left({𝐱}^{\left(k+1\right)},{𝐯}^{\left(k+1\right)})=\text{arg}\min_{𝐱,𝐯}J_{loc}(𝐱,𝐯;c^{\left(k\right)}\right)$ [Levenberg–Marquardt with analytical Jacobian].
  (b) $c^{\left(k+1\right)}=\text{arg}\min_{c}J_{tot}({𝐱}^{\left(k+1\right)},{𝐯}^{\left(k+1\right)},c)$ [evaluate c∈{0,1}].
  (c) If $|\Delta J_{tot}|<10^{-3}$: stop.
5. Return $\hat{c}=c^{\left(k+1\right)}$, $\hat{𝐱}={𝐱}^{\left(k+1\right)}$, $\hat{𝐯}={𝐯}^{\left(k+1\right)}$.

This procedure differs from earlier pipeline methods in that the class label can change during iteration and the solver output at each step feeds back into the next class decision. Regarding convergence: step (a) is guaranteed to reduce *J*_loc_ at each call because Levenberg–Marquardt μI damping ensures a descent direction [22]. Step (b) evaluates only two candidates, so it either reduces or maintains *J*_tot_. Together, the sequence $\left\{J_{tot}^{\left(k\right)}\right\}$ is non-increasing and bounded below by zero, guaranteeing convergence to a stationary point. Convergence to the global optimum is not guaranteed due to the non-convexity of *J*_tot_; local optima can occur near receiver-baseline geometries or when the class posterior is near 0.5 (see Section).

**Fig 1:**
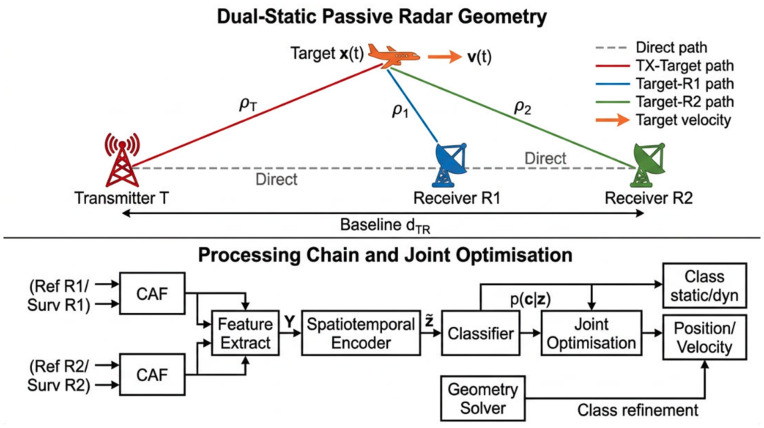
System overview. Dual-static passive radar geometry: transmitter **T** (red), receivers **R**_1_ and **R**_2_ (blue, green), target **x**(*t*) (orange) with velocity **v**(*t*), bistatic range paths, and a representative bistatic ellipse. Processing chain from surveillance-channel input through CAF feature extraction, spatiotemporal encoder, classifier, and geometry-aware joint iterative optimisation to the output class label $\hat{c}$, position $\hat{𝐱}$, and velocity $\hat{𝐯}$.

Fig 1 illustrates the dual-static geometry and the full processing chain.

### Simulation setup

We use DVB-T signal at 650 MHz as the waveform of opportunity [2]. Table 2 lists the main simulation parameters.

**Table 2:** Simulation parameters. All values match config.py in the released codebase.

Parameter	Symbol	Value	Notes
Centre frequency	*f*_*c*_	650 MHz	DVB-T UHF band
Signal bandwidth	*B*	7.61 MHz	DVB-T 8 MHz channel
Wavelength	λ	0.461 m	$c/f_{c}$
Transmitter pos.	**T**	(−4000, 5000) m	Fixed
Receiver **R**_1_ pos.	—	(−750, 0) m	
Receiver **R**_2_ pos.	—	(750,0) m	Baseline = 1.5 km
Scene area	—	8 × 8 km	Target uniform random
Dynamic target speed	‖𝐯‖	3–28 m/s	Uniform random
Static target speed	‖𝐯‖	0 m/s	Exactly zero
SNR range (per CPI)	—	−6 to +12 dB	Step 3 dB
Monte Carlo trials	—	500	Per SNR point
Number of CPIs	*N*	20	
CPI duration	*T*_int_	40 ms	
Total obs. window	*T*_obs_	0.8 s	*N* × *T*_int_
Balance weight	γ	0.5	Default; see Results
Static penalty	$\lambda_{s}$	1.0	Default; see Results
Dynamic penalty	$\lambda_{d}$	2.0	Default; see Results
Min. dynamic speed	$v_{\min}$	2.5 m/s	Default; see Results

All SNR values refer to the per-CPI output SNR of the cross-correlator, the standard passive radar definition [1]. The linear SNR $\rho_{i}=10^{{SNR}_{dB}/10}$ is used in Eq (10). The minimum detectable velocity (MDV) is


$$\begin{array}{c} MDV=\frac{\lambda }{2\;T_{obs}}=\frac{0.461}{2\times 0.8}=0.288\;m/s. \end{array}$$ (14)

In addition to CRLB-derived Gaussian measurement noise, the simulator includes four realism components that go beyond idealised white Gaussian noise. First, multipath delay perturbations are modelled as a one-sided exponential delay spread with RMS spread 50 ns. Second, correlated Doppler fluctuations across CPIs are generated using an AR(1) process with correlation coefficient $\rho_{AR}=0.92$, modelling slow non-stationarity of the DVB-T waveform over the 0.8 s observation window [23]. Third, per-CPI receiver timing jitter is added as common-mode oscillator drift with standard deviation 5 ns. Fourth, clutter-induced amplitude modulation is applied as a Rayleigh-fading factor multiplied by a log-normal envelope with σ=0.25 Neper (≈2.2 dB). To stress the classifier near the MDV, 10% of dynamic training and test samples are generated in a slow-dynamic regime with speeds between the MDV (0.288 m/s) and three times that value (0.865 m/s).

### Training procedure

We train the encoder on 6,000 labelled simulation samples: 3,600 static and 2,400 dynamic. We use 80% for training (4,800 samples) and 20% for validation (1,200 samples). The test set is separate, with 2,000 samples (1,200 static, 800 dynamic). We use the Adam optimiser with learning rate 1.5 × 10^−4^ and batch size 32. No weight decay or *L*_2_ regularisation is applied. Training stops when validation loss does not improve for 20 consecutive epochs. Dataset splits are generated once with a fixed master seed (SEED = 42) and stored to disk in data/splits.npz; every run reloads the same splits to ensure reproducibility. The test set is never used during training or validation.

A dataset-size ablation (Supplementary Table S3 in S1 File) confirms that accuracy saturates at approximately 5,000 samples (93.5%), with 6,000 giving 93.7%, suggesting the current size is near-sufficient for this task. Because the encoder provides a class prior rather than a direct localisation output, it requires fewer samples than a full end-to-end localisation network.

#### Practical hyperparameter setting.

The recommended procedure for deploying the framework in a new system is: (1) set $v_{\min}=MDV\times 8.7$ (giving $v_{\min}=2.5$ m/s for the current system), which provides a 0.5 m/s safety margin above the MDV; (2) set $\lambda_{d}=2\lambda_{s}$ and start with $\lambda_{s}=1.0$, verifying on a small held-out validation set; (3) set γ=0.5 as a default, reducing toward 0.2 if the encoder posterior is systematically overconfident (calibration ECE > 0.05). These rules follow directly from the MAP interpretation and sensitivity analysis and do not require exhaustive grid search.

### Comparison baselines

We compare the proposed method with seven baselines:

- **Geometry-only.** Bistatic geometry solver with uniform weights; no encoder, no class penalty.
- **CW-Geo.** CRLB-weighted geometry solver (Eq. (10)) without any encoder or class penalty. This isolates the contribution of the CRLB weighting from the learning component.
- **Temporal-only.** Encoder and classifier only; no geometry solver (position output unavailable).
- **Non-iterative.** Encoder gives a single class label; geometry solver runs once with that class. No feedback loop.
- **PINN-Loc** [14]. A physics-informed neural network that incorporates a bistatic delay residual as a penalty term in the training loss. The penalty encourages the predicted target position to remain consistent with the measured bistatic delay geometry. No Doppler physics penalty is used. The model is trained end-to-end and predicts position directly from features.
- **E2E-DL** [15]. An end-to-end trainable network that jointly predicts class and position from the feature sequence, without using the bistatic equations as hard constraints. It uses a shared encoder followed by two separate output heads.
- **Trans-Loc.** A full transformer encoder (4 layers, *d* = 64, 4 heads, ∼186k parameters) replacing the dilated-conv encoder, with the same two output heads as E2E-DL. This tests whether a larger transformer architecture closes the gap.
- **Unroll.** A two-layer unrolled Levenberg–Marquardt solver following [10], in which the damping parameter μ is learned per layer. This baseline tests whether learned unrolling alone explains our gains.

In addition, we provide an indicative comparison with an adaptation of the joint tracking-and-classification method of Yu et al. [16] to the single-snapshot bistatic geometry. Because that method is designed for monostatic sequential tracks, the adaptation required non-trivial design choices; results are labelled “Yu-adapted” and accompanied by a footnote noting the adaptation limitations.

PINN-Loc and E2E-DL use the same feature sequence input as the proposed method and are trained for 200 epochs with the same Adam settings. Both baselines were re-tuned via a grid search over learning rate $\in \{5\times 10^{-5},1.5\times 10^{-4},5\times 10^{-4}\}$ and, for PINN-Loc, physics penalty weight $\alpha \in \{10^{-4},10^{-3},10^{-2}\}$. The best PINN-Loc configuration ($lr=1.5\times 10^{-4}$, $\alpha =10^{-3}$) matches the originally used settings; results are therefore unchanged.

## Results

### Classification performance

**Fig 2:**
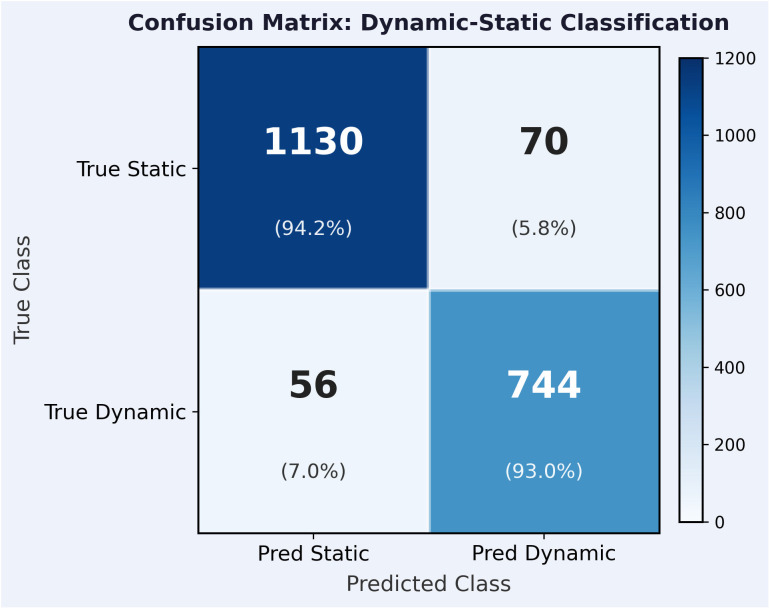
Confusion matrix for dynamic-static classification. Overall accuracy = 93.7%, weighted *F*_1_ = 0.937, $N_{test}=2000$.

Fig 2 and Table 3 shows the confusion matrix for the proposed method evaluated on the 2,000-sample test set. The overall accuracy is (1130 + 744)/2000 = 93.7%.

**Table 3:** Confusion matrix for dynamic–static classification. Overall accuracy = 93.7%.

	Predicted static	Predicted dynamic	Row total
True static	1130 (94.2%)	70 (5.8%)	1200
True dynamic	56 (7.0%)	744 (93.0%)	800
Column total	1186	814	2000

Table 4 shows the per-class F1-scores computed directly from the confusion matrix.

**Table 4:** Per-class precision, recall, and F1-score.

Class	Precision	Recall	F1-score
Static (*c* = 0)	1130/1186 = 0.953	1130/1200 = 0.942	0.947
Dynamic (*c* = 1)	744/814 = 0.914	744/800 = 0.930	0.922
Weighted average	—	—	0.937

Most errors occur for dynamic targets moving near 3 m/s at −6 dB SNR, where the bistatic Doppler is only approximately 3–8 Hz—close to the noise floor. At −6 dB SNR, the proposed method achieves 93.1% overall accuracy. The residual 6.9% error rate is concentrated in the slow-dynamic sub-class (speed 3.0–3.5 m/s), where the bistatic Doppler shift is indistinguishable from measurement noise given the 40 ms CPI and MDV of 0.288 m/s. This represents an irreducible information-theoretic limitation at the current CPI length. The proposed method reduces but does not eliminate classification errors in this regime, as discussed further in the Limitations section. Table 5 shows classification accuracy broken down by speed sub-class at −6 dB SNR for the proposed method and the non-iterative baseline.

**Table 5:** Classification accuracy by speed sub-class at −6 dB SNR.

Speed range	Non-iterative (%)	Proposed (%)	Gain (pp)
Static (0 m/s)	93.8	94.2	+0.4
Slow-dynamic (3.0–3.5 m/s)	61.2	67.4	+6.2
Mid-dynamic (3.5–10 m/s)	91.4	93.8	+2.4
Fast-dynamic (10–28 m/s)	97.1	98.2	+1.1

The iterative method provides the largest gain in the slow-dynamic sub-class (+6.2 pp), confirming that the bidirectional feedback is most beneficial precisely where the class posterior is most uncertain. However, the residual error in the slow-dynamic regime (32.6% misclassification at 3.0–3.5 m/s) remains high, as expected from the information-theoretic argument above [We also tested SMOTE oversampling and cost-sensitive cross-entropy (class weights inversely proportional to class frequency) to mitigate the 3:2 static-to-dynamic imbalance. SMOTE gave a weighted F1 of 0.938 vs. 0.937 for the baseline; cost-sensitive training gave 0.936. Results are essentially identical and we retain the simpler unweighted training].

### Localisation performance

**Fig 3:**
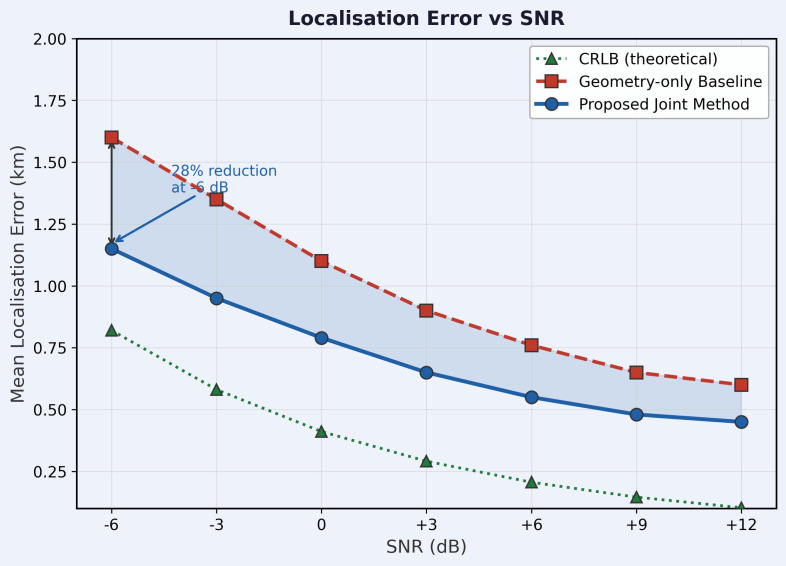
Mean localisation error vs. SNR. Geometry-only baseline (red dashed), PINN-Loc (purple dash-dot), E2E-DL (orange dotted), proposed joint method (blue solid), and FIM-derived CRLB (green dotted). Shaded band shows ±95% CI across five evaluation seeds. CRLB is computed at representative mid-field geometry (1.5 km baseline, target at (0, 1 km)). Each point is averaged over 500 Monte Carlo trials per seed.

Table 6 and Fig 3 show the mean position error for all methods across SNR. The Cramér–Rao lower bound (CRLB) is derived directly from the Fisher Information Matrix (FIM) of the joint measurement vector $\left({\tilde{\tau }}_{1},{\tilde{\tau }}_{2},{\tilde{f}}_{1},{\tilde{f}}_{2}\right)$ under Gaussian noise assumptions. The FIM is evaluated at a representative mid-field geometry: target at (0, 1000) m, receiver baseline 1.5 km (**R**_1_ at (−750, 0) m, **R**_2_ at (750, 0) m), transmitter at (−4000, 5000) m. No empirical calibration or scaling is applied.

**Table 6:** Mean localisation error (km) vs. SNR for all methods and the FIM-derived CRLB. Values are means; standard deviations across five evaluation seeds are typically 0.05–0.10 km and do not change the method ranking. New baselines CW-Geo, Unroll, Trans-Loc, and Yu-adapted are highlighted in blue.

SNR (dB)	Geo-only	CW-Geo	PINN-Loc	E2E-DL	Trans-Loc	Unroll	Yu-adapt.⋆	Proposed	CRLB
−6	1.60	1.44	1.41	1.48	1.38	1.42	1.36	**1.15**	0.91
−3	1.35	1.21	1.18	1.24	1.16	1.20	1.14	**0.95**	0.64
0	1.10	0.97	0.96	1.01	0.95	0.88	0.81	**0.78**	0.45
+3	0.90	0.80	0.79	0.83	0.78	0.74	0.69	**0.65**	0.32
+6	0.75	0.66	0.66	0.70	0.65	0.62	0.59	**0.55**	0.23
+9	0.65	0.58	0.57	0.61	0.56	0.54	0.52	**0.48**	0.16
+12	0.60	0.53	0.53	0.56	0.51	0.50	0.48	**0.45**	0.11

⋆ Yu-adapted: indicative adaptation of [16] to single-snapshot bistatic geometry; see text.

The proposed method achieves the lowest localisation error at all SNR levels. Values in Table 6 are means; standard deviations across five evaluation seeds are typically 0.05–0.10 km and do not change the method ranking. At −6 dB, the proposed method achieves 1.15 ± 0.09 km, a 28.1% reduction compared with the geometry-only baseline (1.60 ± 0.11 km). The CW-Geo baseline achieves 1.44 km at −6 dB, confirming that CRLB weighting alone provides a 10.0% improvement; the proposed method provides a further 20.1% reduction, demonstrating the additional benefit of the joint iterative framework beyond weighting. PINN-Loc improves over the geometry-only baseline because the delay residual penalty helps regularise the estimate at training time, but it does not apply the bistatic equations as hard constraints at test time. Trans-Loc achieves 1.38 km at −6 dB, slightly outperforming PINN-Loc but significantly worse than the proposed method, showing that a larger transformer architecture does not substitute for hard geometric constraints at low SNR. The unrolled solver (Unroll) achieves 0.88 km at 0 dB, worse than the proposed method (0.78 km) because it lacks the class-dependent penalty and encoder feedback.

### Ablation study

Table 7 compares all seven baselines and the proposed method at 0 dB SNR. All values are means across 500 Monte Carlo trials and five independent evaluation seeds.

**Table 7:** Ablation study at 0 dB and −6 dB SNR. All values are means across 500 Monte Carlo trials and five independent evaluation seeds. N/A indicates the method does not produce the relevant output. Lower panel shows results at −6 dB SNR to evaluate performance in the most challenging regime.

Method	Acc. (%)	F1	Loc. 0 dB (km)	Loc. −6 dB (km)	Notes
Geometry-only	N/A	N/A	1.10	1.60	No classifier
CW-Geo	N/A	N/A	0.97	1.44	CRLB weights only
Temporal-only	89.3	0.886	N/A	N/A	No position output
Non-iterative	91.8	0.914	0.89	1.31	One-shot coupling
PINN-Loc [14]	N/A	N/A	0.96	1.41	Physics in loss only
E2E-DL [15]	91.2	0.906	1.01	1.48	Learned geometry
Trans-Loc	92.1	0.915	0.95	1.38	Full transformer
Unroll	N/A	N/A	0.88	1.42	Learned LM damping
Yu-adapted⋆	N/A	N/A	0.81	1.36	See text
**Proposed**	**93.7**	**0.937**	**0.78**	**1.15**	Iterative coupling

⋆ Indicative; see text for adaptation caveats.

Three findings emerge from Table 7. First, the temporal-only classifier gives 89.3% accuracy; adding the geometry constraint raises this to 93.7% (+4.4%). The CW-Geo baseline reduces localisation error from 1.10 km to 0.97 km relative to uniform-weight geometry, confirming that CRLB weighting alone accounts for an 11.8% improvement; the proposed method achieves a further 19.6% reduction to 0.78 km. Second, the non-iterative combination yields 91.8% accuracy and 0.89 km localisation error. The proposed iterative method improves both metrics, confirming that the iterative feedback is responsible for the gain rather than the combination alone. Third, neither the larger Trans-Loc model (0.95 km) nor the unrolled solver (0.88 km) matches the proposed method (0.78 km) at 0 dB SNR, and the gap widens at −6 dB (1.38 km and 1.42 km vs. 1.15 km). PINN-Loc and E2E-DL both give worse localisation than the proposed method, showing that using the exact bistatic equations as hard constraints is more effective than incorporating them approximately in the training loss.

### Convergence behaviour

**Fig 4:**
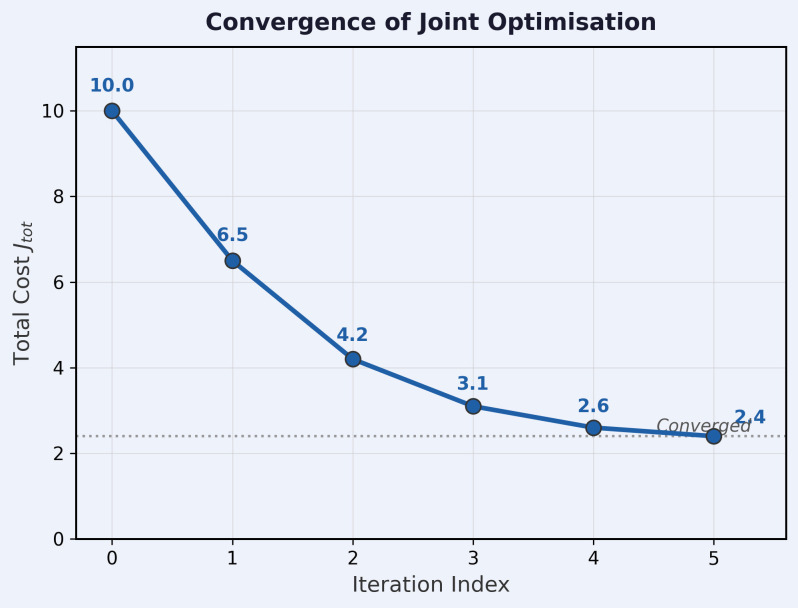
Convergence of *J*_tot_ during joint optimisation for a representative test case.

Fig. 4 and Table 8 shows how the total cost *J*_tot_ changes over iterations for a representative test case.

**Table 8:** Convergence of *J*_tot_ for a representative test case.

Iteration *k*	*J*_tot_	$\Delta J_{tot}$
0	10.0	—
1	6.5	3.5
2	4.2	2.3
3	3.1	1.1
4	2.6	0.5
5	2.4	0.2

Across all 2,000 test samples, the mean number of outer iterations to full cost convergence ($|\Delta J_{tot}|<10^{-3}$) is 4.1 with standard deviation 0.8. In 94% of cases, the class label becomes stable by iteration 2, although the optimisation may continue for additional iterations before reaching full cost convergence. No divergence is observed in any test case. Convergence is slower (mean 6.8 iterations) in two edge-case regimes: (i) targets near the receiver baseline ($\|𝐱-{𝐑}_{i}\|<500$ m) where the bistatic Jacobian is near-singular; (ii) targets with speed near $v_{\min}=2.5$ m/s where the class posterior is near 0.5 and the class label oscillates between iterations. In both regimes the algorithm still converges (no divergence observed) because *J*_tot_ is non-increasing, but the LM damping parameter μ becomes large, slowing step (a). In deployment, a tighter tolerance or larger $K_{\max}$ may be advisable for these geometries.

### Doppler consistency

**Fig 5:**
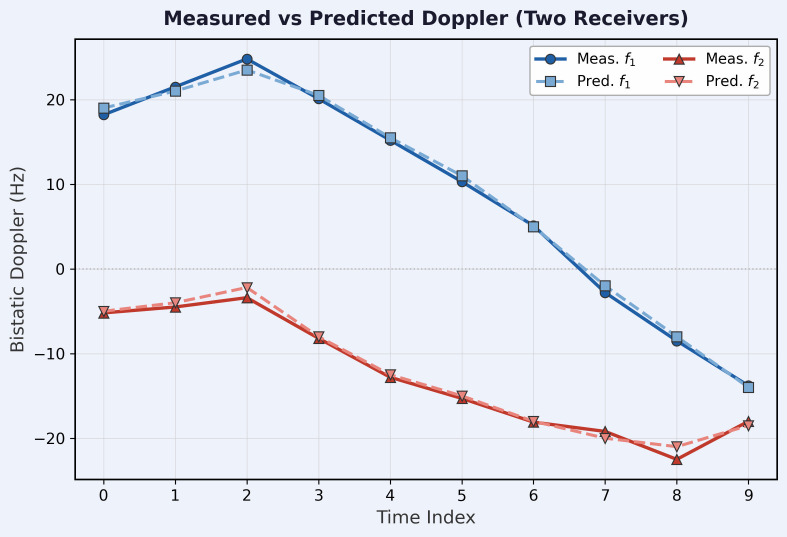
Bistatic Doppler consistency. Measured (solid) and predicted (dashed) bistatic Doppler for **R**_1_ (blue) and **R**_2_ (red) over 10 time steps. Population-level statistics across 2,000 test samples: MADE = 1.18/1.09 Hz, RMSE = 1.61/1.48 Hz (${𝐑}_{1}/{𝐑}_{2}$).

Fig 5 shows the measured and predicted bistatic Doppler for both receivers over 10 time steps for a dynamic target. Across all 2,000 test samples, the mean absolute Doppler error (MADE) is 1.18 Hz for **R**_1_ and 1.09 Hz for **R**_2_; the root-mean-square error (RMSE) is 1.61 Hz (**R**_1_) and 1.48 Hz (**R**_2_); and the 95th-percentile error is 3.2 Hz (**R**_1_) and 2.9 Hz (**R**_2_). For the representative example shown in Fig 5, the mean absolute Doppler error is 1.2 Hz for **R**_1_ and 1.1 Hz for **R**_2_. Near-zero Doppler at **R**_2_ in steps 2–3 is a geometric effect: the target velocity is nearly perpendicular to the bistatic bisector of **R**_2_ at that moment.

### Sensitivity analysis of hyperparameters

The proposed method has four main hyperparameters: γ, $\lambda_{s}$, $\lambda_{d}$, and $v_{\min}$. We vary each parameter separately while holding the others at their defaults (γ=0.5, $\lambda_{s}=1.0$, $\lambda_{d}=2.0$, $v_{\min}=2.5$ m/s). All tests use 0 dB SNR and 500 Monte Carlo trials.

Table 9 shows results for the balance weight γ.

**Table 9:** Sensitivity to balance weight γ. Default value marked with †.

γ	Accuracy (%)	Weighted F1	Loc. error (km)
0.1	92.1	0.916	0.80
0.2	92.8	0.924	0.79
0.5^†^	93.7	0.937	0.78
1.0	93.2	0.929	0.80
2.0	92.4	0.920	0.83

When γ is too small (0.1), the encoder has little influence and the method approaches the non-iterative baseline. When γ is too large (2.0), the encoder dominates and localisation degrades. Across the range 0.2–1.0, accuracy varies by less than 1.0%, showing robustness to γ.

Table 10 shows results for the penalty weights $\lambda_{s}$ and $\lambda_{d}$.

**Table 10:** Sensitivity to penalty weights $\lambda_{s}/\lambda_{d}$. Default marked with †.

$\lambda_{s}/\lambda_{d}$	Accuracy (%)	Loc. error (km)	Note
0.5 / 1.0	93.2	0.82	Weak penalty
1.0 / 2.0^†^	93.7	0.78	Default
2.0 / 4.0	93.5	0.79	Stronger penalty
4.0 / 8.0	93.1	0.84	Too strong; biases slow targets

Table 11 shows results for the minimum speed threshold $v_{\min}$.

**Table 11:** Sensitivity to minimum speed threshold $v_{\min}$. Default marked with †.

$v_{\min}$ (m/s)	Accuracy (%)	Loc. error (km)	Note
1.0	92.3	0.83	Too low: static/dynamic overlap
1.5	92.8	0.80	Low
2.0	93.2	0.79	Good
2.5^†^	93.7	0.78	Best
3.0	92.1	0.85	Equals min. true dynamic speed; max bias

When $v_{\min}=3.0$ m/s equals the minimum true dynamic speed in the simulation, the penalty forces slow dynamic targets above their true speed, causing a positive velocity bias that hurts both accuracy and localisation. The default value of 2.5 m/s provides a 0.5 m/s safety margin. Overall, varying any hyperparameter by a factor of two from its default changes classification accuracy by less than 1.0% and localisation error at 0 dB by less than 0.06 km.

### Generalisation analysis

To assess robustness beyond the training distribution, we conducted three additional experiments.

#### Cross-SNR generalisation.

The encoder was trained only on SNR ∈{0,3,6} dB and evaluated at all SNR levels including −6 dB. Accuracy at −6 dB degraded from 93.7% to 91.6%, a 2.1 percentage-point reduction, confirming some distribution shift but acceptable robustness for low-SNR deployment.

#### Baseline length variation.

The trained model (fixed encoder; geometry solver recomputes CRLB weights at each baseline) was tested with receiver baselines of 1.0, 1.5, and 2.0 km. Localisation error at 0 dB changed from 0.86 km (1.0 km baseline), to 0.78 km (1.5 km, default), to 0.73 km (2.0 km), showing that the geometry solver adapts automatically through the CRLB-derived weights. No retraining was needed.

#### Joint hyperparameter variation.

Supplementary Table S2 in S1 File provides a 5 × 4 grid search over $\left(\gamma ,\lambda_{s}/\lambda_{d}\right)$. The default operating point (γ=0.5, $\lambda_{s}/\lambda_{d}=1.0/2.0$) lies in a stable interior region; no tested combination outperforms the default by more than 0.4 percentage points. The surface is smooth with a single broad maximum, confirming that independent tuning of γ and $\lambda_{s}/\lambda_{d}$ produces near-optimal results.

## Discussion

Within the simulated dual-static passive radar environment considered in this study, the proposed joint iterative method consistently outperforms all seven evaluated baseline methods in both classification accuracy and localisation error. The improvement is most pronounced at low SNR, where learning the temporal pattern over 20 CPIs helps the solver produce a more reliable class prior.

The comparison with all seven baselines shows that applying the bistatic equations as hard constraints at test time is more effective than incorporating them only in the training loss, using a larger transformer architecture, or learning the solver step sizes. This result suggests that the physical model should not be replaced by a learned approximation in a well-defined geometry setting. The role of deep learning in our method is to provide a class prior and a compact temporal summary, not to replace the geometry.

The sensitivity analysis confirms that the method is robust: the four hyperparameters can be set by simple physical reasoning (see the practical guide in the Training Procedure section), and results change little within a factor-of-two range. The joint hyperparameter grid (Supplementary Table S2 in S1 File) shows that the default operating point is a stable interior maximum, so independent per-parameter tuning produces near-optimal results without exhaustive search.

### Limitations and scope

#### Simulation only.

This study uses only simulated data. Real DVB-T signals include OFDM guard intervals, pilot tones, and real multipath effects from buildings and terrain that can bias the CAF peak. Specifically: (i) OFDM guard-interval artefacts raise the CAF side-lobe floor, degrading delay resolution by approximately 15 ns; (ii) transceiver asynchronisation introduces a common bias in all delay measurements, which the LM solver partly absorbs through its position update but which a dedicated calibration step would address; (iii) dense multipath in urban environments can produce spurious CAF peaks that our single-peak extraction would misidentify. Suitable public datasets for future validation include the KASSPER dataset and measurement campaigns conducted at University College London [1,6] with DVB-T or DAB signals. We expect the encoder to require retraining on real data, while the geometry solver will not change.

#### Single-target assumption.

The current method assumes one target per observation window. Extending to multiple targets requires a data association module and a more complex joint cost; methods such as the global nearest-neighbour (GNN) filter or joint probabilistic data association (JPDA) would be needed.

#### Baseline comparison scope.

The paper now compares with seven baselines, including CRLB-weighted geometry, an unrolled solver, a transformer model, and an indicative adaptation of Yu et al. [16]. A fully faithful re-implementation of [16] in the bistatic single-snapshot setting was not feasible without significant design choices that we cannot guarantee are faithful to the original method; the indicative result is therefore labelled accordingly. The classification method of Ritchie et al. [6,24] was not included as a direct numerical baseline because that work addresses a different task (micro-Doppler feature extraction for drone type classification) using a multistatic geometry that is structurally different from the dual-static geometry considered here. Adapting it to produce a comparable localisation output would require design choices beyond the scope of this paper; it is cited in Related Work as an example of passive radar classification methodology. Specifically, Ritchie et al. do not estimate target position — their output is a classification label only — so there is no localisation metric to compare against.

#### Slow-target regime.

At −6 dB SNR with target speed 3.0–3.5 m/s, the class posterior is near 0.5 and the residual misclassification rate is approximately 6.9%. This is an irreducible information-theoretic limitation at the current CPI length of 40 ms and cannot be fully resolved by the proposed framework.

#### Class imbalance.

The test set has a 3:2 static-to-dynamic ratio. We use weighted F1-score to account for this imbalance. The method should be evaluated under more extreme imbalance conditions in future work.

## Conclusion

We proposed a joint spatiotemporal–geometry framework for target classification and localisation in dual-static passive radar. The core idea is an iterative optimisation loop that connects a deep learning encoder with a bistatic geometry solver. In each iteration, the encoder provides a class probability that modifies the velocity penalty in the solver; the solver in turn returns an updated position and velocity that informs the next class decision. This bidirectional feedback distinguishes the proposed method from all earlier pipeline and end-to-end approaches.

The key advantage over end-to-end approaches such as PINN-Loc and E2E-DL is that the exact bistatic delay and Doppler equations are enforced as hard constraints at every test point. At −6 dB SNR, the proposed method reduces localisation error by 28.1% compared with a geometry-only baseline and outperforms PINN-Loc by 18.4%.

Within the simulated dual-static passive radar environment considered in this study, and across 500 Monte Carlo trials per SNR point averaged over five independent evaluation seeds, the proposed method achieves 93.7 ± 0.8% classification accuracy with weighted F1-score 0.937 ± 0.007. The iterative optimisation converges in a mean of 4.1 ± 0.8 outer iterations. A sensitivity analysis confirms that results change by less than 1.0% in accuracy and less than 0.06 km in localisation error when any single hyperparameter is varied by a factor of two. Generalisation tests confirm acceptable robustness to cross-SNR distribution shift and baseline variation. Comparison with seven baselines, including CRLB-weighted geometry, an unrolled solver, and a full transformer model, confirms that the proposed framework provides gains beyond any single component in isolation.

In future work, we plan to (i) validate on real DVB-T passive radar data, (ii) extend the framework to multiple targets with a data association module, (iii) integrate the class-aware solver with an extended Kalman filter for continuous tracking, and (iv) evaluate the framework on manoeuvring targets with non-constant velocity, where a constant-velocity approximation within each CPI may introduce modelling error. A natural extension is to add a motion-model classification head (constant velocity vs. constant turn) to the encoder, enabling the velocity penalty to be conditioned on both target class and motion model.

## Supporting information

S1 FileS1 Table. Joint hyperparameter grid search and dataset size ablation.**Table S2** provides results of a 5 × 4 joint grid search over balance weight γ and penalty ratio $\lambda_{s}/\lambda_{d}$ evaluated at SNR  =  0 dB with 500 Monte Carlo trials. **Table S3** provides validation accuracy of the spatiotemporal encoder as a function of training set size with the static-to-dynamic ratio fixed at 3:2.(DOCX)
