# Correction: A unified spatiotemporal–geometry framework for target classification and localisation in dual-static passive radar

**DOI:** 10.1371/journal.pone.0357583

**Published:** 2026-09-01

**Authors:** Hongmin Wang, Zhiyong Lei, Xing Liu

There is an error in affiliation 1 for author Hongmin Wang. The correct affiliation 1 is: School of Mechatronic Engineering, Xi’an Technological University, Xi’an, Shaanxi, China.
