# Supplementary material for: A unified spatiotemporal–geometry framework for target classification and localisation in dual-static passive radar
Source: PLoS One. 2026 Jun 2;21(6):e0350515. doi: 10.1371/journal.pone.0350515 (PMC13229372; doi:10.1371/journal.pone.0350515)
Supplement: S1 File — Table S2 provides results of a 5 × 4 joint grid search over balance weight γ and penalty ratio λs/λd evaluated at SNR = 0 dB with 500 Monte Carlo trials. Table S3 provides validation accuracy of the spatiotemporal encoder as a function of training set size with the static-to-dynamic ratio fixed at 3:2. (DOCX) [file pone.0350515.s001.docx]

\section*{Supporting Information} \paragraph*{S1~Table.} {\bf Joint hyperparameter grid search and dataset size ablation.} Table~S2 provides results of a $5 \times 4$ joint grid search over balance weight $\gamma$ and penalty ratio $\lambda_s/\lambda_d$ evaluated at SNR\,=\,0\,dB with 500 Monte Carlo trials. Table~S3 provides validation accuracy of the spatiotemporal encoder as a function of training set size with the static-to-dynamic ratio fixed at 3:2.
